# Decreased microRNA-155 in Behcet’s disease leads to defective control of autophagy thereby stimulating excessive proinflammatory cytokine production

**DOI:** 10.1186/s13075-021-02517-8

**Published:** 2021-05-06

**Authors:** Liang Liang, Qingyun Zhou, Lujia Feng

**Affiliations:** The First Affiliated Hospital of Chongqing Medical University, Chongqing Key Lab of Ophthalmology, Chongqing Eye Institute, Chongqing Branch of National Clinical Research Center for Ocular Diseases, Chongqing, P. R. China

**Keywords:** Behcet’s disease, Autophagy, MicroRNA-155

## Abstract

**Background:**

Earlier, we reported that the microRNA (miR)-155 expression in dendritic cells (DCs) from Behcet’s disease (BD) patients was decreased and affected cytokine production of DCs. In this study, we investigated the mechanisms whereby miR-155 regulates cytokine production by DCs.

**Methods:**

The formation of autophagosomes in DCs was detected by transmission electron microscopy. Western blotting was used to detect the protein levels of LC3, Beclin-1, P62, p-mTOR, and p-Akt in DCs. TNF-α, IL-6, and IL-1β expression were investigated by ELISA. MiR-155 mimics were transfected to DCs to evaluate its effects on autophagy and cytokine production. RNA interference was used to downregulate the expression of TAB2.

**Results:**

The formation of autophagosomes was found in DCs of active BD patients. The expressions of LC3-II, Beclin-1, and P62 were significantly increased in DCs of active BD patients compared to that of inactive BD patients and healthy controls. The expressions of IL-6, IL-1β, and TNF-α were significantly increased in DCs of active BD patients compared to that of healthy controls. The autophagy promoter (3-MA) and inhibitor (rapamycin) significantly decreased or increased the expression of TNF-α, IL-6, and IL-1β by DCs. The expression of LC3-II and Beclin-1 was significantly increased, but the expression of P62 proteins was decreased in DCs transfected with miR-155 mimics or after TAB2 was downregulated. The expression of TNF-α, IL-6, and IL-1β was decreased in DCs after miR-155 was upregulated or TAB2 was downregulated. The ratios of p-Akt/Akt and p-mTOR/mTOR were decreased in DCs after miR-155 was upregulated.

**Conclusions:**

These results suggest that miR-155 affects the production of TNF-α, IL-6, and IL-1β by DCs through activation of the Akt/mTOR signaling pathway and by affecting the process of autophagy.

## Introduction

Behcet’s disease (BD) is a major uveitis entity in China, which symptoms include skin lesions, genital ulcers, oral aphthae, and recurrent uveitis [1–3]. BD is now considered as an autoinflammatory disease. Many studies have demonstrated that dendritic cells (DCs) play a key role in modulating the aberrant immune reaction during the development of BD [4–7]. Earlier research from our group showed that the expression of miR-155 in DCs of BD patients was decreased [8]. However, the exact mechanism of how miR-155 regulates the function of DCs in patients with BD is still not completely elucidated and was therefore the subject of the study reported here, whereby we focused on the possible role of autophagy.

Autophagy is an evolutionarily catabolic pathway that degrades abnormal proteins, damaged organelles, and recycles cellular components [9]. This process is initiated by a double-membraned autophagosome, in which the autophagosomes fuse with the lysosomes, resulting in their degradation by acidic hydrolases. Lysosomes subsequently release the end-products of autophagic digestion into the cytoplasm, which then participate in cellular metabolism [10]. Autophagy has been shown to play a crucial role in maintaining normal intracellular homeostasis of eukaryotic cells and the dysfunction of autophagy may be involved in the pathogenesis of various diseases, including cancer, inflammation, neurodegenerative disease, and autoimmune disease [11–15]. Our earlier studies showed that autophagy-related gene (ATGs) polymorphisms were associated with the development of BD [16]. MiR-155 can modulate the process of autophagy and participates in the development of various diseases [17–19]. In this study, we show that autophagy was activated in DCs from active BD patients, but that autophagic degradation was decreased. We furthermore show that autophagy was involved in the miR-155-dependent regulation of cytokine release from DCs.

## Materials and methods

### Subjects

Twenty-three active BD patients (13 males and 10 females, with an average age of 36.5 years), 8 inactive BD patients (5 males and 3 females, with an average age of 37.1 years) and 28 healthy individuals (16 males and 12 females, with an average age of 34.4 years) were included in this study (some patients were included in two or three experiments) (Table 1). All subjects were enrolled between July 2015 and November 2020. The BD patients were diagnosed according to the criteria of the International Study Group for Behcet’s Disease [20]. None of the active BD patients enrolled in this study were taking immunosuppressive agents when first visiting our hospital. The inactive BD patients were enrolled after complete control of the intraocular inflammation and termination of all medications for at least 6 months. The healthy controls had no clinical history of systemic diseases or uveitis. Venous blood samples were taken from patients and controls. Written consent was collected from each healthy control and BD patient. This study obeyed the tenets of the Declaration of Helsinki and was approved by our Clinical Ethical Research Committee.

**Table 1:** Clinical parameters

	Active BD patients (23)	Inactive BD patients (8)	Normal controls (28)	*P* value
Age	36±2	37±5	34±3	*P*>0.05
Gender (male/female)	13/10	5/3	16/12	*P*>0.05

Values are expressed as mean±SD

### Cell culture

Peripheral blood mononuclear cells (PBMCs) and CD14^+^ monocytes were separated from the peripheral blood and purified according to the methods described earlier [21]. Immature monocyte-derived DCs were obtained as described earlier [22]. DCs were treated with autophagy activator (rapamycin (100nM, Sigma-Aldric, St Louis, MO, USA) or inhibitor (3-MA(10mM, Sigma-Aldrich, St Louis, MO, USA) together with LPS (Sigma-Aldrich, St Louis, MO, USA) for 24 h.

### Transmission electron microscopy

The collected DCs were prepared according to the methods described earlier [23]. An H-7500 transmission electron microscope (Hitachi, Japan) was used to identify autophagosome-like vesicles at 80 kV.

### Western blotting

Western blotting was performed following methods described earlier [8]. All the band detection is within the linear range.

### MiRNAs and siRNAs transfection

The miR-155 mimics from GenePharma (Shanghai, China) were transfected into DCs according to methods described previously [8]. The siTAB2-RNAi-LV and pGC-FU-RNAi-NC-LV as controls were from GeneChem (Shanghai, China) and transfected into DCs according to the user’s manual.

### ELISA

The concentrations of TNF-α, IL-6, and IL-1β in cell supernatants were measured by ELISA (Human DuoSet ELISA Development Kit; R&D Systems, Minneapolis, MN).

### Statistical analysis

One-way ANOVA and Student’s *t* test were carried out by using SPSS17.0 software (SPSS Inc., Chicago, IL, USA). *P* values < 0.05 were considered significant.

## Results

### Autophagy is activated, but the capability of autophagic degradation is decreased in DCs from active BD patients

**Fig. 1:**
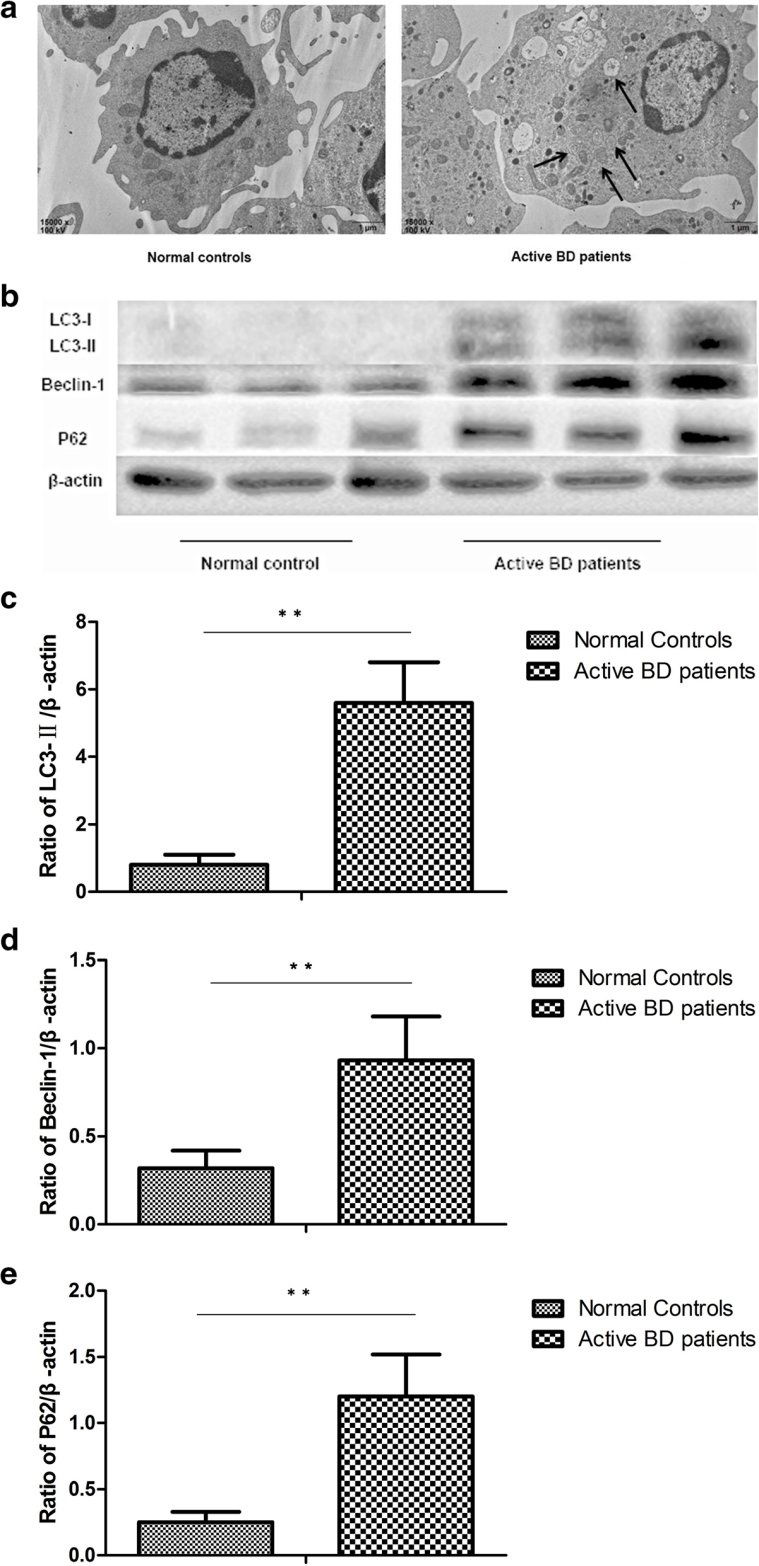
Autophagy is involved in the cytokine production of DCs. **a** Representative TEM photomicrographs of DCs from normal controls (*n*=3), inactive BD patients (*n*=3), and active BD patients (*n*=3). **b** Expression levels of LC3-I, LC3-II, Beclin-1, and P62 protein in DCs from normal controls (*n*=8), inactive BD patients (*n*=8), and active BD patients (*n*=8) were quantified by Western blot. **c**–**e** Quantification of expression levels of LC3-II, Beclin-1, and P62 proteins. *p<0.05, **p<0.01

The most important manifestation of autophagy is the formation of autophagosomes and this was investigated by TEM. Our results showed that autophagosomes were only found in DCs from active BD patients, but not in DCs from inactive BD patients and healthy controls (Fig. 1a). To further identify whether autophagy was activated in patients with active BD, the expression of Beclin-1 and LC3, which are two important markers of autophagy, were investigated by Western blot. The results showed that compared with inactive BD patients and healthy controls, and the protein levels of Beclin-1 and LC3-II were significantly increased in DCs from active BD patients after stimulation with LPS (Fig. 1b–d). There was no difference concerning the expression of LC3 and Beclin-1 between the inactive BD patients and healthy controls. Taken together, these results suggested that autophagy was activated in DCs from active BD patients.

P62/SQSTM1 (hereafter referred to as P62), serving as anautophagic substrate, is degraded during autophagy [24]. P62 accumulates in cells when degradation capacity via autophagy is impaired. We next investigated the expression of P62 in DCs from active BD patients. The results showed that the protein expression level of P62 was significantly higher in DCs of active BD patients than that of inactive BD patients and healthy controls, which indicates that autophagic degradation was decreased in active BD patients, despite the presence of a large number of autophagosomes (Fig. 1b, e).

### Autophagy is involved in cytokine production of DCs

**Fig. 2:**
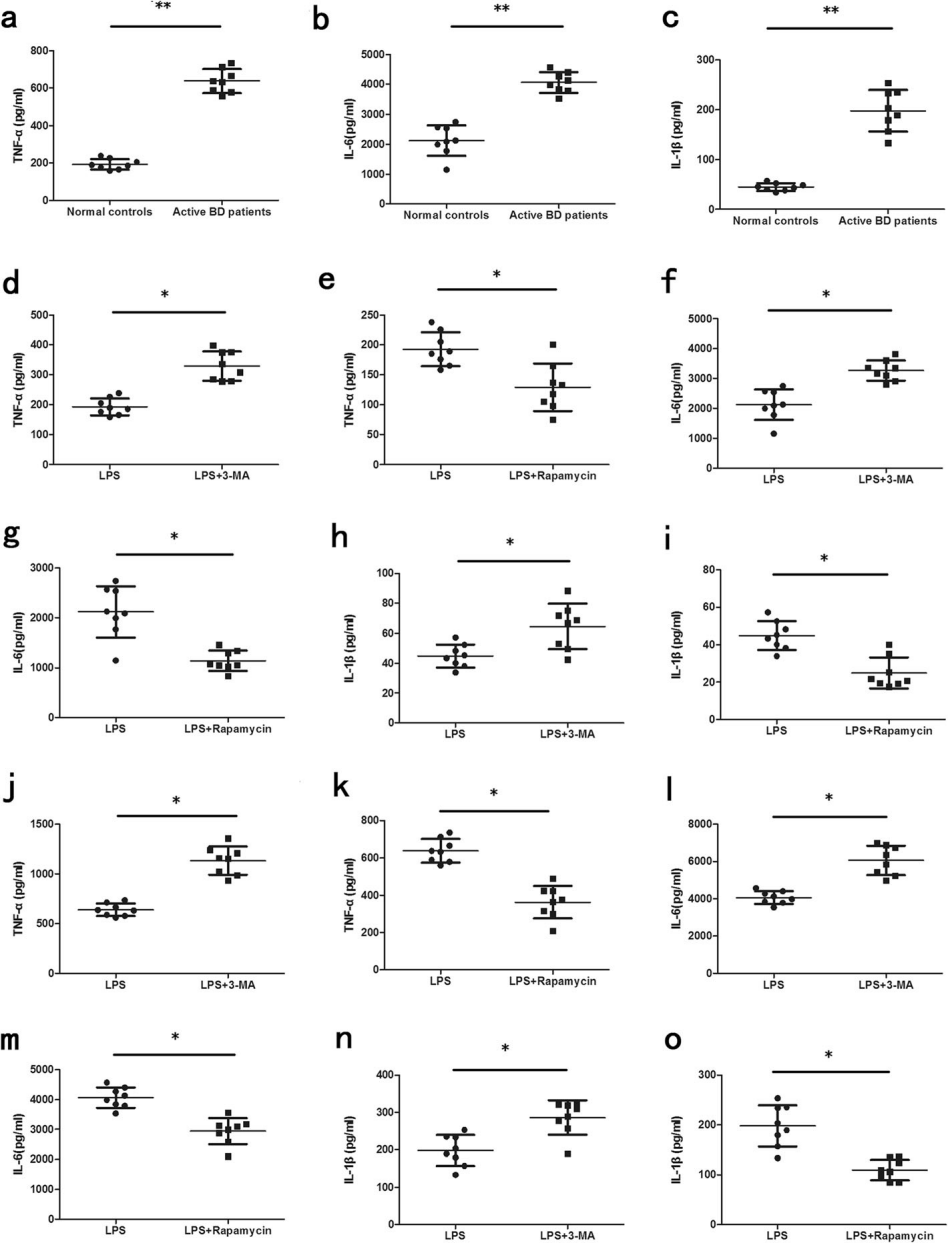
Autophagy is involved in the cytokine production of DCs. DCs from normal controls (*n*=8) and active BD patients (*n*=8) were assessed. **a**–**c** DCs were stimulated with LPS (100ng/ml) for 24 h. Cytokines including TNF-α, IL-1β, and IL-6 were measured in the culture supernatants by ELISA. **d**–**i** DCs from the normal controls were treated with 3-MA (10mM) or rapamycin (100nM) together with LPS (100ng/ml) for 24 h. TNF-α, IL-1β, and IL-6 were measured in the culture supernatants by ELISA. **j**–**o** DCs from active BD patients were treated with 3-MA (10mM) or rapamycin (100nM) together with LPS (100ng/ml) for 24 h. TNF-α, IL-1β, and IL-6 were measured in the culture supernatants by ELISA. *p<0.05, **p<0.01

In view of the dysfunctional autophagy in DCs of active BD patients, further experiments were performed to examine whether autophagy had an effect on cytokine production by DCs. The results demonstrated that the expression of TNF-α, IL-6, and IL-1β of LPS-treated DCs from active BD patients was significantly higher than that of healthy controls (Fig. 2a–c). It has been proven that 3-MA could inhibit autophagy through PI3K pathway, and rapamycin could activate autophagy by mTOR pathway [25, 26]. The results showed that 3-MA and rapamycin, which are the inhibitor or promoter of autophagy, significantly increased or decreased the expression levels of TNF-α, IL-6, and IL-1β by DCs treated with LPS (Fig. 2d–o). These data suggest that autophagy plays a role as a negative regulator of inflammation by regulating the expression of inflammatory cytokines.

### MiR-155 is involved in the cytokine production of DCs induced by dysfunctional autophagy

**Fig. 3:**
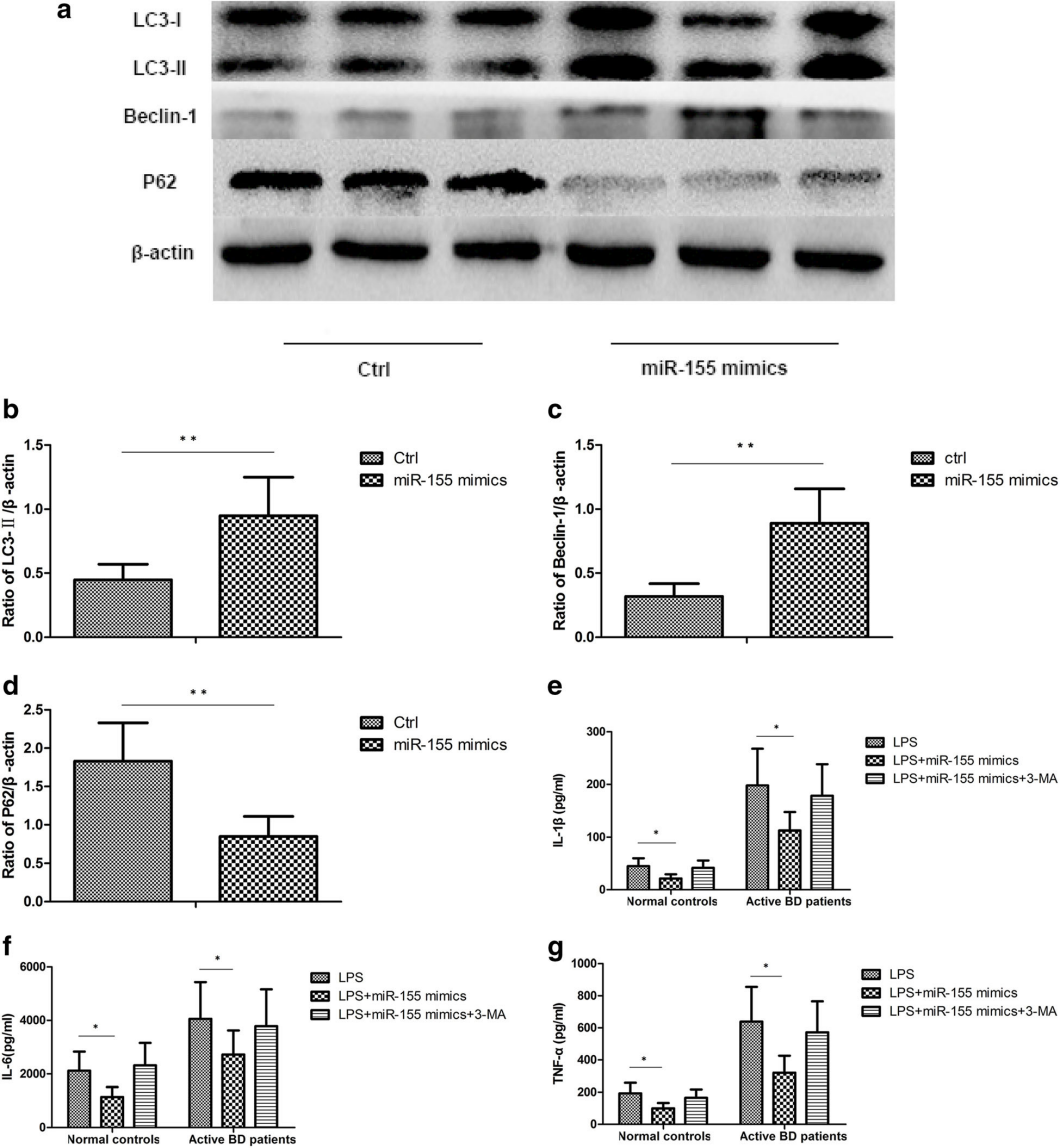
MiR-155 is involved in the cytokine production of DCs induced by dysfunctional autophagy. DCs from normal controls (*n*=8) and active BD patients (*n*=8) were assessed. DCs were transfected with control mimics and miR-155 mimics at a final concentration of 100 nM. After 48 h, DCs were stimulated with 100ng/mL LPS for 24 h. **a** Expression levels of LC3-I, LC3-II, Beclin-1, and P62 protein in DCs from normal controls and active BD patients were quantified by Western blot. **b**–**d** Quantification of expression levels of LC3-II, Beclin-1, and P62 proteins. **e**–**g** TNF-α, IL-1β, and IL-6 were measured in the culture supernatants by ELISA. *p<0.05, **p<0.01

Earlier, we showed that the expression of miR-155 was decreased in DCs from active BD patients [8]. Moreover, miR-155 has been reported to be involved in autophagy regulation [17, 27–29]. A further experiment was carried out to investigate if miR-155 was associated with cytokine production by DCs induced by dysfunctional autophagy. The results showed that in DCs overexpressing miR-155, the expression of LC3-II and Beclin-1 was significantly increased after stimulation with LPS. The expression of P62 was significantly decreased in DCs overexpressing miR-155 after stimulation with LPS (Fig. 3a–d). As compared with DCs transfected with the control sequence, TNF-α, IL-6, and IL-1β production were significantly decreased in DCs overexpressing miR-155. However, there was no difference concerning the expression of TNF-α, IL-6, and IL-1β in LPS-treated DCs overexpressing miR-155 after treatment with 3-MA compared to DCs transfected with the control sequence (Fig. 3e–g).

### TAB2 is involved in the effects of MiR-155 on autophagy

**Fig. 4:**
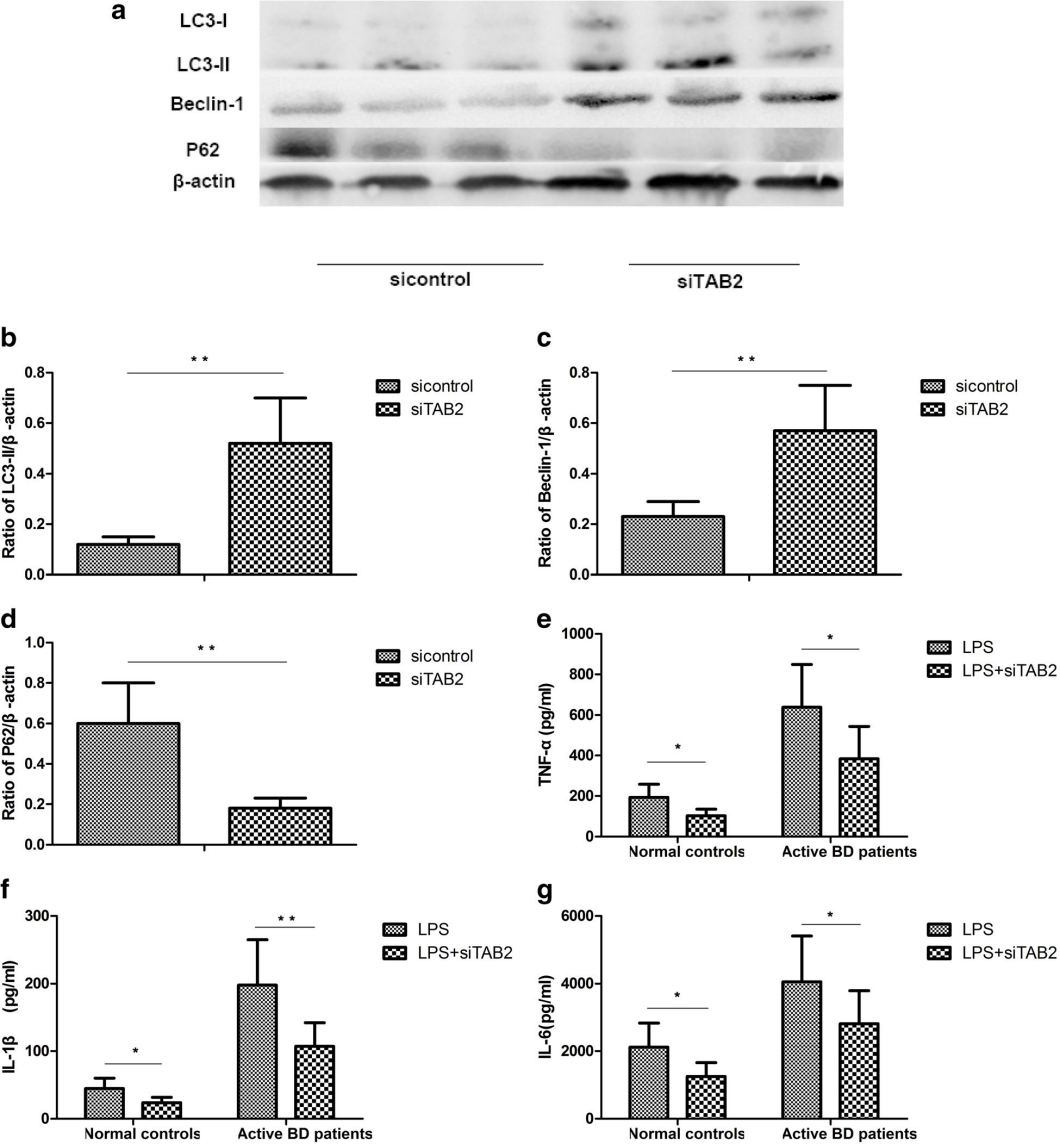
TAB2 is involved in the effects of miR-155 on autophagy. DCs from normal controls (*n*=8) and active BD patients (*n*=8) were assessed. DCs were transfected with siRNA or control siRNA and then stimulated with 100ng/mL LPS for 24 h. **a** Expression levels of LC3-I, LC3-II, Beclin-1, and P62 protein in DCs from the normal controls and active BD patients were quantified by Western blot. **b**–**d** Quantification of expression levels of LC3-II, Beclin-1, and P62 proteins. **e**–**g** TNF-α, IL-1β, and IL-6 were measured in the culture supernatants by ELISA. *p<0.05, **p<0.01

Transforming growth factor β-activated kinase 1-binding protein 2 (TAB2) is the direct target of miR-155. The expression of TAB2 in DCs from active BD patients is increased, and its expression can be suppressed by miR-155 [8]. Since the above findings showed that miR-155 was involved in the cytokine production of DCs induced by autophagy, a further experiment was performed to investigate whether TAB2 was involved in the effects of miR-155 on autophagy. The results showed that the expression of LC3-II and Beclin-1 was significantly increased, but that the expression of P62 was decreased in DCs after TAB2 was downregulated (Fig. 4a–d). The expression of TNF-α, IL-6, and IL-1β by DCs treated with LPS was significantly decreased after TAB2 was downregulated (Fig. 4e–g).

### MiR-155 regulates autophagy through Akt/mTOR pathway

**Fig. 5:**
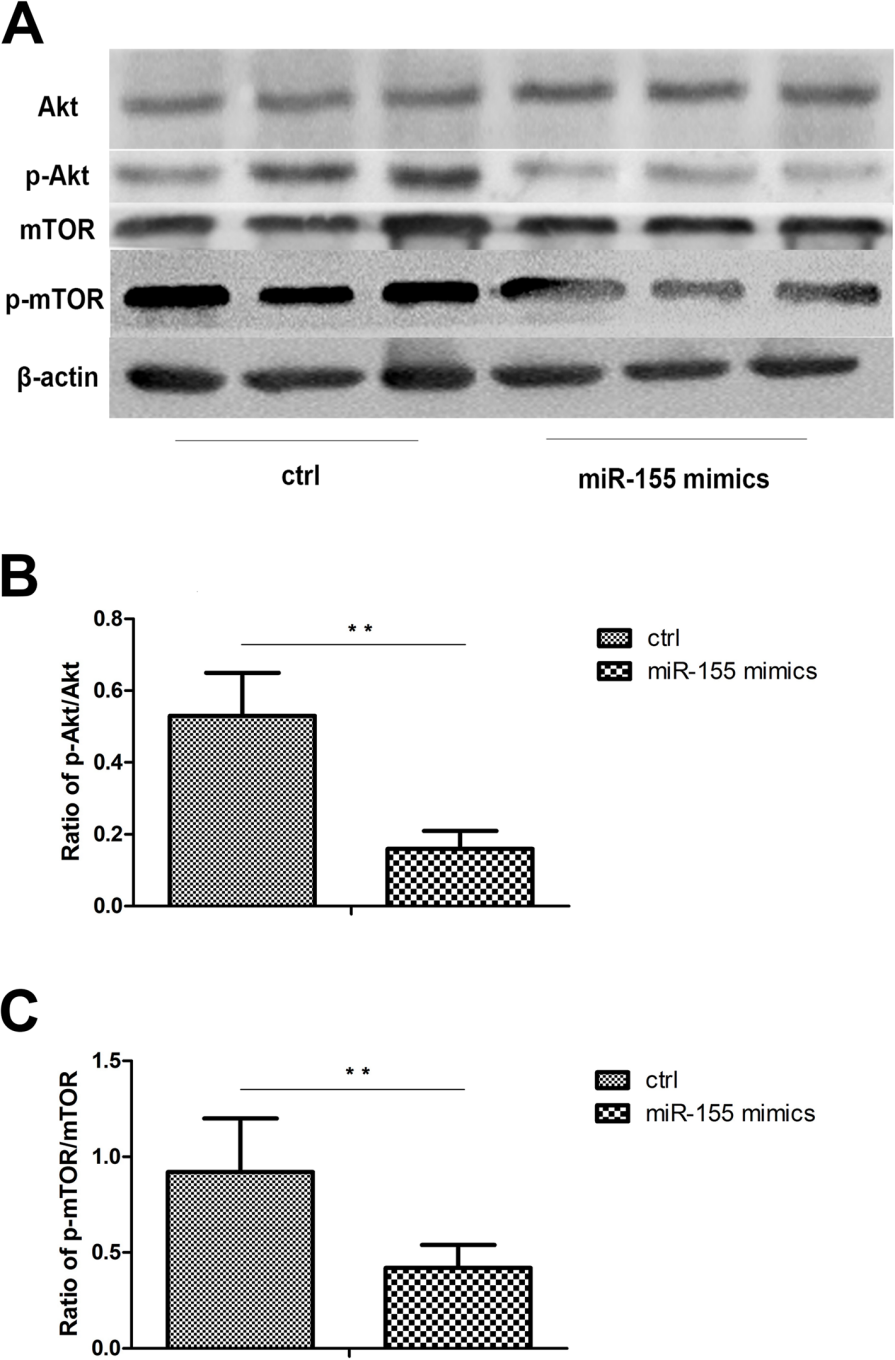
MiR-155 regulates autophagy through Akt/mTOR pathway. DCs from normal controls (*n*=8) and active BD patients (*n*=8) were assessed. After 48 h, DCs were stimulated with 100ng/mL LPS for 24 h. **a** Total levels of Akt and mTOR, together with phosphorylation levels of Akt and mTOR in DCs were determined by Western blotting. **b**–**c** Quantification of expression levels of p-Akt/Akt and p-mTOR/mTOR proteins. *p<0.05, **p<0.01

The aforementioned results showed that miR-155 could regulate cytokine production of DCs by controlling autophagy. The Akt/mTOR pathway is the most common signaling pathway involved in autophagy. We further examined whether miR-155 exerted its effects on autophagy by activating the Akt/mTOR pathway. The results showed that the ratios of p-Akt/Akt and p-mTOR/mTOR were significantly increased in DCs treated with LPS after transfection with miR-155 mimics (Fig. 5a–c). These results suggested that miR-155 may activate autophagy via the Akt/mTOR signaling pathway.

## Discussion

In this study, we found that the autophagy process was activated in DCs from active BD patients. Autophagic degradation was however decreased, which, in turn, results in proinflammatory cytokine release by DCs. Moreover, we found that miR-155 and its target protein TAB2 were involved in the proinflammatory cytokine production of DCs by regulating autophagy through the Akt/mTOR signaling pathway. These results indicate that miR-155 might take part in the development of BD by controlling autophagy. In other words, our findings indicate that certain triggers in a genetically predisposed individual stimulates autophagosome formation, whereby a defective control of the subsequent degradation of autophagic products leads to a local stimulation of proinflammatory cytokine release.

It has been reported that autophagy was associated with the development of animal uveitis models [30, 31]. However, the role of autophagy in the pathogenesis of human uveitis is still unknown. In this study, we first detected the activity level of autophagy in DCs from active and inactive BD patients. The results showed that the autophagosomes were found in DCs from active BD patients. Furthermore, we found that as compared with inactive BD patients and healthy controls, the protein levels of two important markers of autophagy activity, Beclin-1 and LC3-II, were significantly increased in DCs derived from active BD patients. Additionally, the protein expression level of P62 was significantly increased in DCs of active BD patients as compared with inactive BD patients and healthy controls, which indicated that there is an increased number of autophagosomes but a concurrent buildup of autophagic degradation products.

In view of the impaired autophagic flux in DCs of active BD patients, we further examined if the activity of autophagy is associated with the cytokine production by DCs. Our results showed that compared with healthy controls, the production of TNF-α, IL-6, and IL-1β by DCs from active BD patients stimulated with LPS were significantly increased. We also showed that the autophagy inhibitor (3-MA) or promoter (rapamycin) could upregulate or downregulate the production of TNF-α, IL-6, and IL-1β. These results proved that the autophagy activity level correlated with the production of cytokines by DCs. These results are consistent with previous studies showing that IL-1β, IL-17, and IL-18 levels were downregulated in murine DCs after autophagy was inhibited [32]. Others showed that the production of IL-6, IFN-β, and TNF-α by DCs were decreased after Beclin-1 was knocked out [33]. These results indicate that a defective control of autophagy may trigger cytokine production by DCs.

Earlier, we reported that the expression of miR-155 was downregulated in DCs obtained from active BD patients and was involved in cytokine production by DCs [8]. It has also been reported that the expression of miR-155 was increased or decreased in various diseases and was involved in the development of these diseases by regulating autophagy [17, 19, 34, 35]. In view of the aforementioned reports, we next investigated whether miR-155 exerted its effects on cytokine production by DCs via the process of autophagy. To answer this question, an experiment with miR-155 mimic transfection was performed. The results showed that an miR-155 mimic could promote the expression of LC3-II and Beclin-1, which meant that autophagy activity was increased. More importantly, the protein level of P62 was downregulated in DCs after miR-155 mimic was transfected, showing that autophagic degradation was increased. Additionally, miR-155 mimic transfection reduced the production of TNF-α, IL-1β, and IL-6. The autophagy inhibitor, 3-MA, was able to counteract the effects of miR-155 on cytokine production. All these results suggested that autophagy was involved in the effects of miR-155 on cytokine production by DCs.

TAB2 has been reported to be the target of miR-155, and its protein expression level was elevated in DCs derived from active BD patients [8]. It also been reported that TAB2 could bind to Beclin1 or ATG13 to regulate autophagy [36–38]. A further experiment was performed to investigate whether miR-155 regulates the process of autophagy through TAB2. The results showed that downregulation of TAB2 could promote the autophagic flux. The expression of TNF-α, IL-6, and IL-1β was decreased after TAB2 was downregulated. Taken together, these data indicate that miR-155 regulates autophagy by controlling the expression of TAB2. Our results are in agreement with previous studies that showed that miR-155 promoted autophagy via a decrease in TAB2 expression, thereby stimulating osteoclast formation [17].

The Akt/mTOR pathway is one of the most common signaling pathways involved in the control of autophagy [39, 40]. Rapamycin, as the inhibitor of mTOR, has been shown to alleviate inflammation of the retina [41]. It has also been reported that the use of this mTOR inhibitor was safe and effective in treating non-infectious uveitis [42–44]. In this study, we found that miR-155 could downregulate the phosphorylation level of mTOR and Akt in DCs.

The limitation of our research is that we did not investigate the function of autophagy in other types of uveitis and the detailed mechanisms on how a decreased autophagic degradation results in increased cytokine production. Further experiments are needed to explore the effects of treatment on the function of autophagy in DCs and if the dysfunction of autophagy is a common phenomenon of uveitis.

## Conclusions

Collectively, these results show that miR-155 can control the production of cytokines by DCs via the process of autophagy. Our study provides a greater depth of understanding about the mechanism on how miR-155 is involved in the development of BD and provides evidence for the use of autophagy as a potential therapeutic target of uveitis.

## Data Availability

The datasets used and/or analyzed during the current study are available from the corresponding author on reasonable request.

## Abbreviations

miR: MicroRNA
BD: Behcet’s disease
DCs: Dendritic cells
ATGs: Autophagy-related gene
PBMCs: Peripheral blood mononuclear cells
TAB2: Transforming growth factor β-activated kinase 1-binding protein 2
